# Supplementary material for: Left-right side-specific endocrine signaling complements neural pathways to mediate acute asymmetric effects of brain injury
Source: eLife. 2021 Aug 10;10:e65247. doi: 10.7554/eLife.65247 (PMC8354641; doi:10.7554/eLife.65247)
Supplement: Figure 1—source code 1. [file elife-65247-fig1-code1.zip › Figure 1DE - source code/PA 9 9 4 4 RD Nikolay RepAveraged Prone.docx]

MPA, p_Asym, p_Contra for 9 9 4 4 RD Nikolay task (Prone, ~~Supine~~)

[Daniil.Sarkisyan@farmbio.uu.se](mailto:Daniil.Sarkisyan@farmbio.uu.se)

2019-08-13

## Read PA data

PA are averaged, even if six PA replications were recorded separately. |PA| > mpa.threshold are defined as “asymmetric”. Position = Decubitus = <https://medical-dictionary.thefreedictionary.com/decubitus+position>

mpa.threshold <- 1 # assymetry means >1mm
my.seed <- 20190811 # reproducible Bayessian fit

myname <- "PA 9 9 4 4 RD Nikolay RepAveraged Prone"
# myname <- "PA 9 9 4 4 RD Nikolay RepAveraged Supine"
dtaAve <- read.xlsx("PA_Measurements/Nikolay-SpinoCorticalLesions-2017-06-12.xlsx", sheet="Data", colNames=TRUE) # PA_Measurements/

dtaAve <- dtaAve %>% as_tibble %>%
 filter(Decubitus == "Ventral") %>% # on the belly = Prone = ventral decubitus
 # filter(Decubitus == "Dorsal") %>% # on the spine = Suppine = dorsal decubitus
 transmute( RatID = factor(RatID),
 Decubitus = factor(Decubitus, c("Ventral", "Dorsal"), c("Prone", "Supine")),
 Time = factor(Time, c("30m", "1h", "3h"), c("30min", "60min", "180min") ),
 Trt = factor(Trt, c("Sham", "Ablation"), c("SS", "BI")),
 OS = factor(TrtSide, c("Left", "Right")), # Left only
 PA = PA ) %>%
 mutate( Sym = factor(abs(PA) < mpa.threshold, c(TRUE,FALSE), c("Sym", "Asym"), c("Sym","Asym")),
 Flex = factor( ifelse(Sym=="Sym", NA, ifelse(PA>0,"RF","LF")), c("RF","LF") ),
 Lat = factor( ifelse((OS=="Left" & Flex=="LF") | (OS=="Right" & Flex=="RF"),"Ipsi", "Contra"),
 c("Ipsi","Contra") ) ) %>%
 ungroup %>% droplevels(.)
#dtaAve %>% mutate_if(is.character, as.factor) %>% summary # misspelled levels --> NA

The group sizes are (distinct rats only, same rats with different stimulation locations are counted as duplicates)

| Decubitus | OS | Trt | 30min | 60min | 180min |
| --- | --- | --- | --- | --- | --- |
| Prone | Left | SS | 4 | 4 | 4 |
| Prone | Left | BI | 9 | 9 | 9 |
| Prone | Right | SS | 4 | 4 | 4 |
| Prone | Right | BI | 9 | 9 | 9 |

## PA: Student Model

Prior predictive check

fName <- paste0("PA_SampledModels/", myname,".RData")
#load(fName) #SpeedUp# fit once to load everafter
std.prior <- brm(
 data = dtaAve, family = student,
 PA ~ 0 + OS:Time:Trt,
 prior = c(prior(student_t(3, 0, 10), class = "sigma"),
 prior(normal(0, 3), class = "b")),
 seed = my.seed, chains=my.cores, cores=my.cores, iter=4e4, sample_prior = "only" )

## Compiling the C++ model

## Start sampling

me <- marginal_effects(std.prior, "Trt", method="fitted", conditions=make_conditions(dtaAve, vars = c("OS","Time")))
print(me[[1]])

## Trt OS Time cond__ PA effect1__
## 1 SS Left 30min OS = Left & Time = 30min -0.1698718 SS
## 2 BI Left 30min OS = Left & Time = 30min -0.1698718 BI
## 3 SS Left 60min OS = Left & Time = 60min -0.1698718 SS
## 4 BI Left 60min OS = Left & Time = 60min -0.1698718 BI
## 5 SS Left 180min OS = Left & Time = 180min -0.1698718 SS
## 6 BI Left 180min OS = Left & Time = 180min -0.1698718 BI
## 7 SS Right 30min OS = Right & Time = 30min -0.1698718 SS
## 8 BI Right 30min OS = Right & Time = 30min -0.1698718 BI
## 9 SS Right 60min OS = Right & Time = 60min -0.1698718 SS
## 10 BI Right 60min OS = Right & Time = 60min -0.1698718 BI
## 11 SS Right 180min OS = Right & Time = 180min -0.1698718 SS
## 12 BI Right 180min OS = Right & Time = 180min -0.1698718 BI
## estimate__ se__ lower__ upper__
## 1 -0.0060797668 2.988785 -5.928995 5.844291
## 2 0.0151364501 3.017477 -5.884520 5.888875
## 3 -0.0037914078 2.981710 -5.875517 5.810790
## 4 0.0086138983 3.003142 -5.882452 5.874382
## 5 -0.0047776371 2.989127 -5.836011 5.810545
## 6 -0.0083588589 2.974778 -5.828168 5.840503
## 7 0.0155772025 3.002664 -5.916813 5.874953
## 8 0.0009159328 3.026139 -5.895259 5.903672
## 9 0.0041137519 3.001577 -5.884869 5.880662
## 10 0.0068326181 2.987398 -5.782663 5.866138
## 11 -0.0034107396 2.999109 -5.809563 5.847722
## 12 0.0169984483 3.014092 -5.896233 5.901980

plt[["pa prior vs data"]] <- plot(me, points = TRUE, point_args = list(width = 0.1,shape="o",color="red"), plot=FALSE)[[1]] +
 geom_hline(yintercept=0, linetype="dashed", color="black")
print(plt[["pa prior vs data"]])


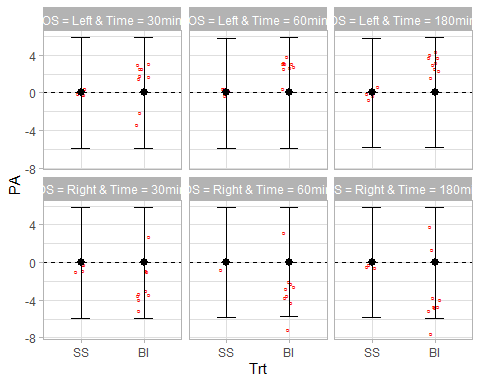


set.seed(1) # reproducible pp_check
tryCatch(print( plt[["PA prior pp_check"]] <- pp_check(std.prior, nsamples = 11) ), error=function(e) "pp_check() failed")


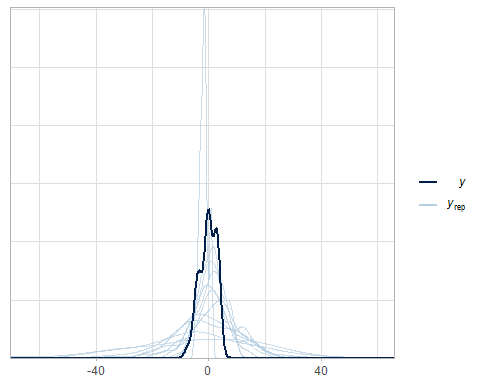


Model fit and QC:

## The desired updates require recompiling the model

## Compiling the C++ model

## Start sampling

## Family: student
## Links: mu = identity; sigma = identity; nu = identity
## Formula: PA ~ 0 + OS:Time:Trt
## Data: dtaAve (Number of observations: 78)
## Samples: 3 chains, each with iter = 40000; warmup = 20000; thin = 1;
## total post-warmup samples = 60000
##
## Population-Level Effects:
## Estimate Est.Error l-95% CI u-95% CI Rhat
## OSLeft:Time30min:TrtSS -0.01 0.31 -0.62 0.62 1.00
## OSRight:Time30min:TrtSS -0.57 0.34 -1.24 0.10 1.00
## OSLeft:Time60min:TrtSS 0.24 0.33 -0.43 0.87 1.00
## OSRight:Time60min:TrtSS 0.02 0.35 -0.73 0.68 1.00
## OSLeft:Time180min:TrtSS -0.17 0.38 -0.90 0.62 1.00
## OSRight:Time180min:TrtSS -0.29 0.30 -0.88 0.31 1.00
## OSLeft:Time30min:TrtBI 2.22 0.34 1.55 2.87 1.00
## OSRight:Time30min:TrtBI -3.26 0.32 -3.85 -2.55 1.00
## OSLeft:Time60min:TrtBI 2.99 0.22 2.53 3.42 1.00
## OSRight:Time60min:TrtBI -2.87 0.38 -3.67 -2.17 1.00
## OSLeft:Time180min:TrtBI 3.27 0.33 2.61 3.90 1.00
## OSRight:Time180min:TrtBI -4.49 0.31 -5.06 -3.82 1.00
## Bulk_ESS Tail_ESS
## OSLeft:Time30min:TrtSS 68426 37753
## OSRight:Time30min:TrtSS 73080 41291
## OSLeft:Time60min:TrtSS 72914 41108
## OSRight:Time60min:TrtSS 69349 38001
## OSLeft:Time180min:TrtSS 69401 39344
## OSRight:Time180min:TrtSS 67768 38113
## OSLeft:Time30min:TrtBI 70412 46239
## OSRight:Time30min:TrtBI 63477 31683
## OSLeft:Time60min:TrtBI 69144 41293
## OSRight:Time60min:TrtBI 68905 44581
## OSLeft:Time180min:TrtBI 72281 45514
## OSRight:Time180min:TrtBI 67497 42256
##
## Family Specific Parameters:
## Estimate Est.Error l-95% CI u-95% CI Rhat Bulk_ESS Tail_ESS
## sigma 0.60 0.12 0.40 0.87 1.00 32259 40441
## nu 1.53 0.37 1.04 2.42 1.00 31673 22469
##
## Samples were drawn using sampling(NUTS). For each parameter, Eff.Sample
## is a crude measure of effective sample size, and Rhat is the potential
## scale reduction factor on split chains (at convergence, Rhat = 1).

## PA: Estimated model marginals

Estimated model medians +- 95% HPDCI (HPDCI = highest posterior density continuous interval) as a table:

emm <- emmeans(std.fit, ~ OS * Trt * Time, transform = "response")
emm_show(emm)

| OS | Trt | Time | emmean | lower.HPD | upper.HPD | p.value |
| --- | --- | --- | --- | --- | --- | --- |
| Left | SS | 30min | -0.021 | -0.617 | 0.624 | 1.00e+00 |
| Right | SS | 30min | -0.572 | -1.229 | 0.113 | 6.83e-01 |
| **Left** | **BI** | **30min** | **2.226** | **1.572** | **2.883** | **9.42e-10** |
| **Right** | **BI** | **30min** | **-3.285** | **-3.890** | **-2.615** | **0.00e+00** |
| Left | SS | 60min | 0.248 | -0.419 | 0.889 | 9.99e-01 |
| Right | SS | 60min | 0.042 | -0.696 | 0.710 | 1.00e+00 |
| **Left** | **BI** | **60min** | **2.991** | **2.549** | **3.440** | **0.00e+00** |
| **Right** | **BI** | **60min** | **-2.849** | **-3.646** | **-2.158** | **8.63e-13** |
| Left | SS | 180min | -0.181 | -0.925 | 0.590 | 1.00e+00 |
| Right | SS | 180min | -0.294 | -0.881 | 0.316 | 9.91e-01 |
| **Left** | **BI** | **180min** | **3.277** | **2.636** | **3.920** | **0.00e+00** |
| **Right** | **BI** | **180min** | **-4.508** | **-5.054** | **-3.823** | **0.00e+00** |

Plot of medians +- 95% HPDCI:


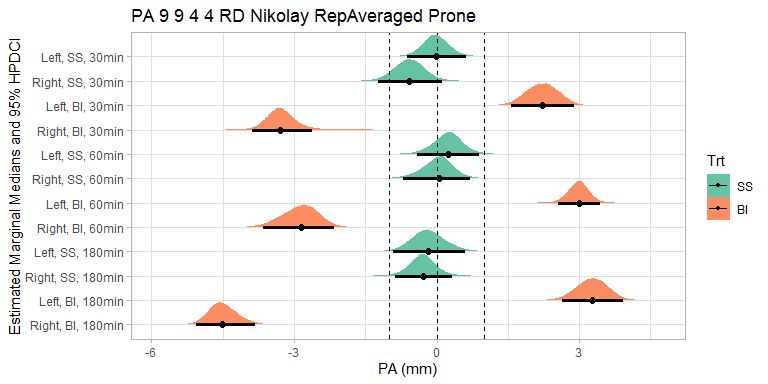


## PA: Contrasts

Contrasts BI vs SS as a table:

emmc <- rbind( contrast(emm, simple="Trt", method = "revpairwise"),
 contrast(emm, simple="OS", method = "revpairwise"),
 contrast(emm, simple="Time", method = "consec") )
emm_show(emmc)

| OS | Time | Trt | contrast | estimate | lower.HPD | upper.HPD | p.value |
| --- | --- | --- | --- | --- | --- | --- | --- |
| **Left** | **30min** | **.** | **BI - SS** | **2.243** | **1.331** | **3.137** | **1.89e-05** |
| **Right** | **30min** | **.** | **BI - SS** | **-2.703** | **-3.593** | **-1.733** | **2.16e-07** |
| **Left** | **60min** | **.** | **BI - SS** | **2.745** | **1.980** | **3.568** | **5.24e-11** |
| **Right** | **60min** | **.** | **BI - SS** | **-2.885** | **-3.905** | **-1.879** | **2.68e-07** |
| **Left** | **180min** | **.** | **BI - SS** | **3.451** | **2.440** | **4.436** | **8.89e-11** |
| **Right** | **180min** | **.** | **BI - SS** | **-4.208** | **-5.028** | **-3.322** | **0.00e+00** |
| . | 30min | SS | Right - Left | -0.557 | -1.478 | 0.344 | 9.56e-01 |
| **.** | **30min** | **BI** | **Right - Left** | **-5.499** | **-6.379** | **-4.524** | **0.00e+00** |
| . | 60min | SS | Right - Left | -0.214 | -1.208 | 0.705 | 1.00e+00 |
| **.** | **60min** | **BI** | **Right - Left** | **-5.841** | **-6.761** | **-5.021** | **0.00e+00** |
| . | 180min | SS | Right - Left | -0.115 | -1.092 | 0.825 | 1.00e+00 |
| **.** | **180min** | **BI** | **Right - Left** | **-7.771** | **-8.665** | **-6.863** | **0.00e+00** |
| Left | . | SS | 60min - 30min | 0.264 | -0.649 | 1.147 | 1.00e+00 |
| Left | . | SS | 180min - 60min | -0.418 | -1.374 | 0.608 | 9.98e-01 |
| Right | . | SS | 60min - 30min | 0.606 | -0.384 | 1.545 | 9.55e-01 |
| Right | . | SS | 180min - 60min | -0.329 | -1.211 | 0.624 | 1.00e+00 |
| Left | . | BI | 60min - 30min | 0.763 | -0.016 | 1.573 | 5.81e-01 |
| Left | . | BI | 180min - 60min | 0.286 | -0.506 | 1.056 | 9.99e-01 |
| Right | . | BI | 60min - 30min | 0.416 | -0.623 | 1.335 | 9.99e-01 |
| **Right** | **.** | **BI** | **180min - 60min** | **-1.637** | **-2.562** | **-0.618** | **2.15e-02** |

Contrasts BI vs SS as a median +- 95% HPDCI (HPDCI = highest posterior density continuous interval):


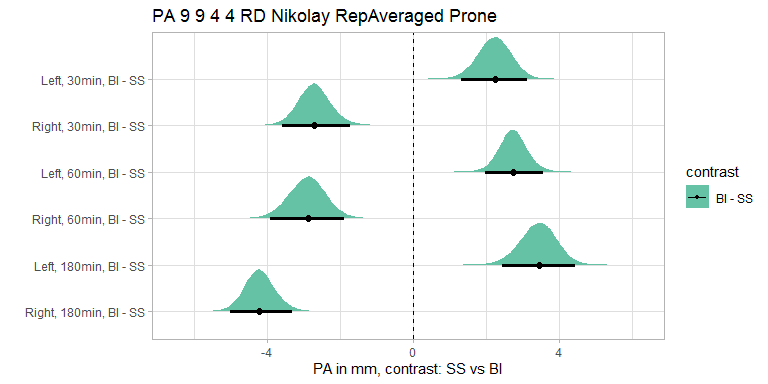


Contrasts “between time” as a median +- 95% HPDCI (HPDCI = highest posterior density continuous interval):


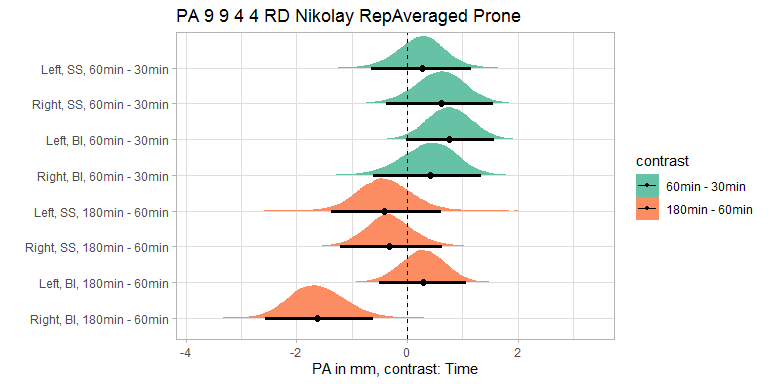


Contrasts Left vs Right treatment side as a median +- 95% HPDCI (HPDCI = highest posterior density continuous interval):


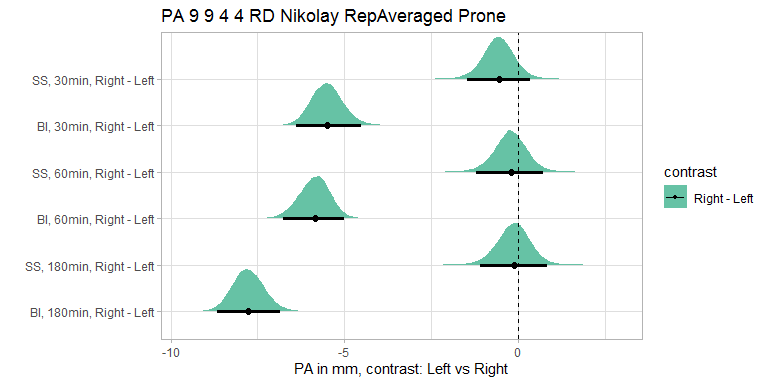


## PA: MCMC conversion diagnostics

Posterior predictive check

set.seed(2) # reproducible pp_check
tryCatch(print( plt[["PA pp_check"]] <- pp_check(std.fit, nsamples = 11) ), error=function(e) "pp_check() failed")


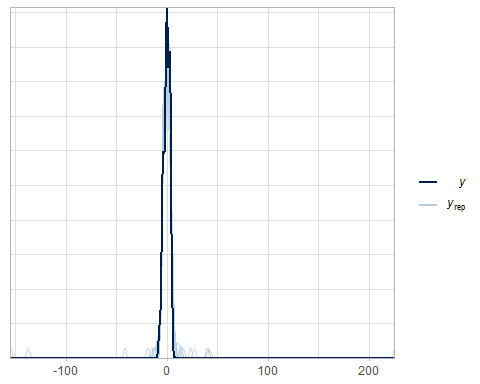


Autocorrelations

print( plt[["MPA ac"]] <- stan_ac(std.fit$fit) )

## 'pars' not specified. Showing first 10 parameters by default.


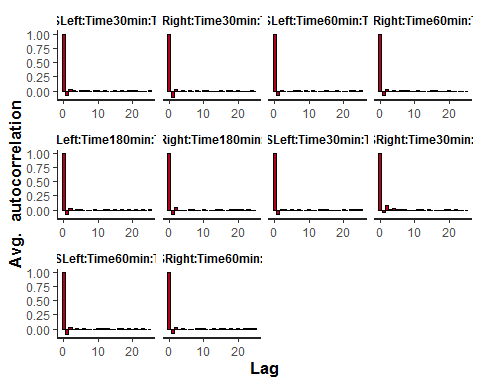


## p_Asym: Model Overview

The data points for “Asymmetric Flexion”

| Decubitus | OS | Trt | Sym | 30min | 60min | 180min |
| --- | --- | --- | --- | --- | --- | --- |
| Prone | Left | SS | Sym | 4 | 4 | 4 |
| Prone | Left | BI | Asym | 9 | 8 | 9 |
| Prone | Left | BI | Sym |  | 1 |  |
| Prone | Right | SS | Sym | 3 | 4 | 4 |
| Prone | Right | SS | Asym | 1 |  |  |
| Prone | Right | BI | Sym | 2 |  |  |
| Prone | Right | BI | Asym | 7 | 9 | 9 |

Model fit and QC:

m.sym <- brm(
 data = dtaAve, family = bernoulli,
 Sym ~ 0 + OS:Trt:Time,
 prior = c(prior(normal(0, 1), class = "b")),
 seed = my.seed, chains=my.cores, cores=my.cores, iter=4e4, sample_prior = "yes")

## Compiling the C++ model

## Start sampling

save(std.prior,std.fit, m.sym, file=fName)

summary(m.sym)

## Family: bernoulli
## Links: mu = logit
## Formula: Sym ~ 0 + OS:Trt:Time
## Data: dtaAve (Number of observations: 78)
## Samples: 3 chains, each with iter = 40000; warmup = 20000; thin = 1;
## total post-warmup samples = 60000
##
## Population-Level Effects:
## Estimate Est.Error l-95% CI u-95% CI Rhat
## OSLeft:TrtSS:Time30min -1.09 0.77 -2.66 0.37 1.00
## OSRight:TrtSS:Time30min -0.53 0.73 -2.01 0.89 1.00
## OSLeft:TrtBI:Time30min 1.64 0.67 0.40 3.02 1.00
## OSRight:TrtBI:Time30min 0.84 0.60 -0.29 2.05 1.00
## OSLeft:TrtSS:Time60min -1.09 0.77 -2.64 0.35 1.00
## OSRight:TrtSS:Time60min -1.09 0.76 -2.63 0.35 1.00
## OSLeft:TrtBI:Time60min 1.21 0.63 0.04 2.50 1.00
## OSRight:TrtBI:Time60min 1.63 0.67 0.39 3.03 1.00
## OSLeft:TrtSS:Time180min -1.09 0.76 -2.62 0.35 1.00
## OSRight:TrtSS:Time180min -1.10 0.76 -2.63 0.34 1.00
## OSLeft:TrtBI:Time180min 1.64 0.67 0.39 3.02 1.00
## OSRight:TrtBI:Time180min 1.64 0.68 0.38 3.04 1.00
## Bulk_ESS Tail_ESS
## OSLeft:TrtSS:Time30min 131072 43152
## OSRight:TrtSS:Time30min 129097 42751
## OSLeft:TrtBI:Time30min 127658 42489
## OSRight:TrtBI:Time30min 127849 43774
## OSLeft:TrtSS:Time60min 138414 44066
## OSRight:TrtSS:Time60min 134613 43782
## OSLeft:TrtBI:Time60min 132611 42794
## OSRight:TrtBI:Time60min 133529 44052
## OSLeft:TrtSS:Time180min 136000 45377
## OSRight:TrtSS:Time180min 135505 44898
## OSLeft:TrtBI:Time180min 128019 42844
## OSRight:TrtBI:Time180min 129734 41686
##
## Samples were drawn using sampling(NUTS). For each parameter, Eff.Sample
## is a crude measure of effective sample size, and Rhat is the potential
## scale reduction factor on split chains (at convergence, Rhat = 1).

## p_Asym: Estimated model marginals

Estimated model medians +- 95% HPDCI (HPDCI = highest posterior density continuous interval) as a table:

emm <- emmeans(m.sym, ~ OS*Trt*Time, transform = "response")

## Warning in model.matrix.default(trms, m, contrasts.arg = contr): variable
## 'Sym' is absent, its contrast will be ignored

emm_show(emm)

| OS | Trt | Time | response | lower.HPD | upper.HPD | p.value |
| --- | --- | --- | --- | --- | --- | --- |
| **Left** | **SS** | **30min** | **0.255** | **0.041** | **0.544** | **6.41e-01** |
| **Right** | **SS** | **30min** | **0.372** | **0.102** | **0.684** | **3.12e-01** |
| **Left** | **BI** | **30min** | **0.834** | **0.633** | **0.969** | **0.00e+00** |
| **Right** | **BI** | **30min** | **0.694** | **0.452** | **0.904** | **3.87e-07** |
| **Left** | **SS** | **60min** | **0.255** | **0.048** | **0.549** | **6.38e-01** |
| **Right** | **SS** | **60min** | **0.254** | **0.046** | **0.546** | **6.35e-01** |
| **Left** | **BI** | **60min** | **0.767** | **0.539** | **0.941** | **4.22e-11** |
| **Right** | **BI** | **60min** | **0.833** | **0.632** | **0.972** | **0.00e+00** |
| **Left** | **SS** | **180min** | **0.254** | **0.048** | **0.548** | **6.27e-01** |
| **Right** | **SS** | **180min** | **0.254** | **0.046** | **0.544** | **6.24e-01** |
| **Left** | **BI** | **180min** | **0.833** | **0.631** | **0.968** | **0.00e+00** |
| **Right** | **BI** | **180min** | **0.834** | **0.630** | **0.969** | **0.00e+00** |

Plot of medians +- 95% HPDCI:


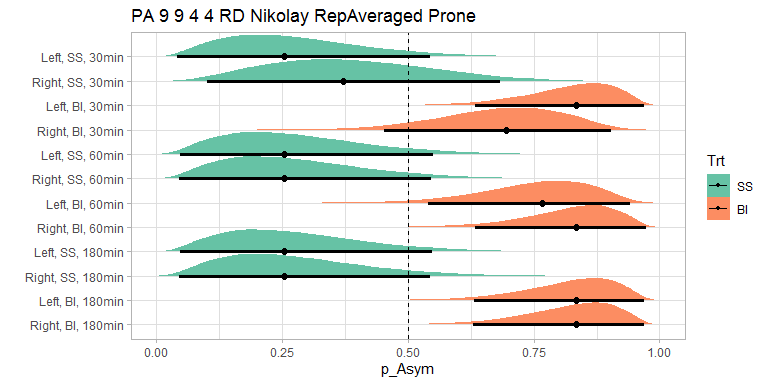


## p_Asym: Contrasts

Contrasts as a table:

emmc <- rbind( contrast(emm, simple="Trt", method = "revpairwise"),
 contrast(emm, simple="OS", method = "revpairwise"),
 contrast(emm, simple="Time", method = "consec") )
emm_show(emmc)

| OS | Time | Trt | contrast | estimate | lower.HPD | upper.HPD | p.value |
| --- | --- | --- | --- | --- | --- | --- | --- |
| **Left** | **30min** | **.** | **BI - SS** | **0.561** | **0.211** | **0.840** | **1.10e-02** |
| Right | 30min | . | BI - SS | 0.311 | -0.081 | 0.673 | 8.20e-01 |
| **Left** | **60min** | **.** | **BI - SS** | **0.495** | **0.132** | **0.802** | **7.16e-02** |
| **Right** | **60min** | **.** | **BI - SS** | **0.560** | **0.213** | **0.840** | **1.04e-02** |
| **Left** | **180min** | **.** | **BI - SS** | **0.560** | **0.211** | **0.838** | **1.05e-02** |
| **Right** | **180min** | **.** | **BI - SS** | **0.562** | **0.214** | **0.838** | **9.60e-03** |
| . | 30min | SS | Right - Left | 0.110 | -0.299 | 0.522 | 1.00e+00 |
| . | 30min | BI | Right - Left | -0.132 | -0.444 | 0.160 | 9.96e-01 |
| . | 60min | SS | Right - Left | -0.001 | -0.394 | 0.383 | 1.00e+00 |
| . | 60min | BI | Right - Left | 0.062 | -0.209 | 0.362 | 1.00e+00 |
| . | 180min | SS | Right - Left | -0.000 | -0.386 | 0.388 | 1.00e+00 |
| . | 180min | BI | Right - Left | 0.001 | -0.269 | 0.265 | 1.00e+00 |
| Left | . | SS | 60min - 30min | 0.000 | -0.382 | 0.401 | 1.00e+00 |
| Left | . | SS | 180min - 60min | -0.000 | -0.393 | 0.383 | 1.00e+00 |
| Right | . | SS | 60min - 30min | -0.109 | -0.509 | 0.307 | 1.00e+00 |
| Right | . | SS | 180min - 60min | -0.001 | -0.394 | 0.377 | 1.00e+00 |
| Left | . | BI | 60min - 30min | -0.062 | -0.357 | 0.212 | 1.00e+00 |
| Left | . | BI | 180min - 60min | 0.062 | -0.210 | 0.360 | 1.00e+00 |
| Right | . | BI | 60min - 30min | 0.131 | -0.165 | 0.439 | 9.96e-01 |
| Right | . | BI | 180min - 60min | 0.000 | -0.264 | 0.270 | 1.00e+00 |

Contrasts BI vs SS as a median +- 95% HPDCI (HPDCI = highest posterior density continuous interval):


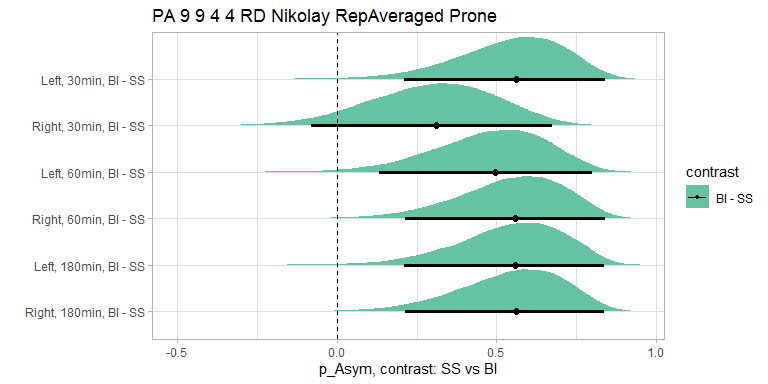


Contrasts “between Time” as a median +- 95% HPDCI (HPDCI = highest posterior density continuous interval):


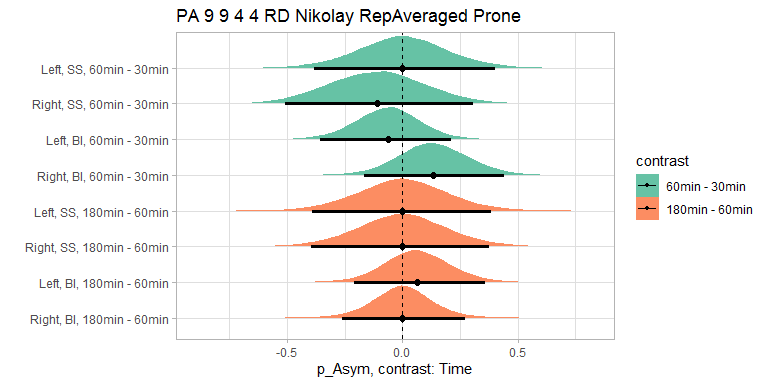


Contrasts Left vs Right treatment side as a median +- 95% HPDCI (HPDCI = highest posterior density continuous interval):


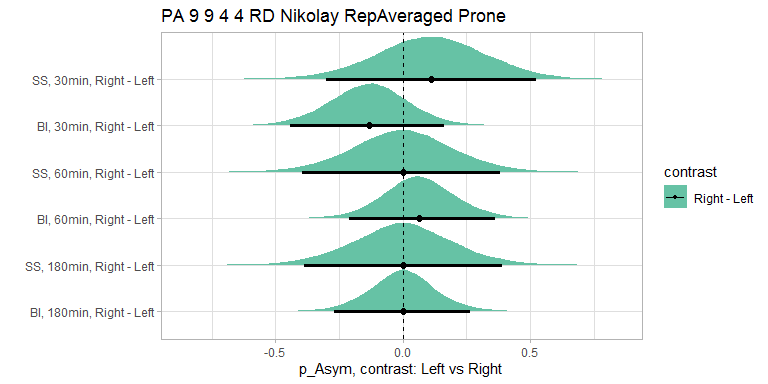


## p_Asym: MCMC conversion diagnostics

Posterior predictive check

set.seed(1) # reproducible pp_check
tryCatch(print( plt[["p_Asym pp_check"]] <- pp_check(m.sym, nsamples = 11) ), error=function(e) "pp_check() failed")


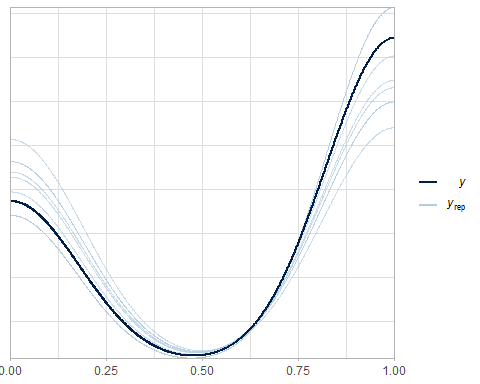


Autocorrelations

print( plt[["p_Asym ac"]] <- stan_ac(m.sym$fit) )

## 'pars' not specified. Showing first 10 parameters by default.


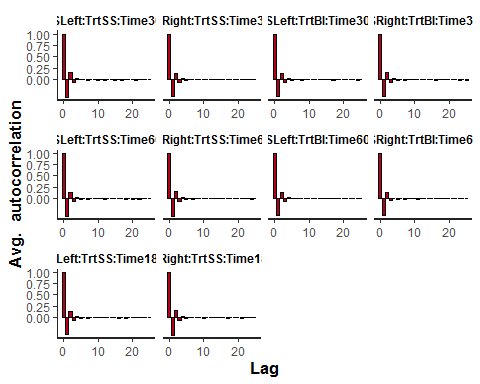


## p_Contra: Model Overview

We add a reference 50/50 level to “ContraLateral Flexion” data: nContra contralateral flexions out on nTrials asymmetric rats.

## Warning in bind_rows_(x, .id): binding character and factor vector,
## coercing into character vector

| Trt | counts | 30min | 60min | 180min |
| --- | --- | --- | --- | --- |
| 50/50 | nContra | 8 | 8 | 9 |
| 50/50 | nTrials | 16 | 16 | 18 |
| BI | nContra | 13 | 16 | 16 |
| BI | nTrials | 16 | 17 | 18 |

Model fit and QC:

m.lat <- brm(
 data = dta5050, family = binomial,
 nContra | trials(nTrials) ~ 0 + Trt:Time,
 prior = c(prior(normal(0, 1), class = "b")),
 seed = my.seed, chains=my.cores, cores=my.cores, iter=4e4, sample_prior = "yes")

## Compiling the C++ model

## Start sampling

## Warning: There were 2 transitions after warmup that exceeded the maximum treedepth. Increase max_treedepth above 10. See
## http://mc-stan.org/misc/warnings.html#maximum-treedepth-exceeded

## Warning: Examine the pairs() plot to diagnose sampling problems

save(std.prior,std.fit, m.sym, m.lat, file=fName)

summary(m.lat)

## Family: binomial
## Links: mu = logit
## Formula: nContra | trials(nTrials) ~ 0 + Trt:Time
## Data: dta5050 (Number of observations: 6)
## Samples: 3 chains, each with iter = 40000; warmup = 20000; thin = 1;
## total post-warmup samples = 60000
##
## Population-Level Effects:
## Estimate Est.Error l-95% CI u-95% CI Rhat Bulk_ESS
## Trt50D50:Time30min -0.00 0.45 -0.89 0.88 1.00 76380
## TrtBI:Time30min 1.12 0.51 0.16 2.17 1.00 72169
## Trt50D50:Time60min 0.00 0.46 -0.90 0.90 1.00 77182
## TrtBI:Time60min 1.75 0.57 0.71 2.93 1.00 76505
## Trt50D50:Time180min -0.00 0.43 -0.85 0.85 1.00 75296
## TrtBI:Time180min 1.50 0.53 0.52 2.58 1.00 73611
## Tail_ESS
## Trt50D50:Time30min 43862
## TrtBI:Time30min 41050
## Trt50D50:Time60min 45323
## TrtBI:Time60min 42379
## Trt50D50:Time180min 44045
## TrtBI:Time180min 42626
##
## Samples were drawn using sampling(NUTS). For each parameter, Eff.Sample
## is a crude measure of effective sample size, and Rhat is the potential
## scale reduction factor on split chains (at convergence, Rhat = 1).

## p_Contra: Estimated model marginals

Estimated model medians +- 95% HPDCI (HPDCI = highest posterior density continuous interval) as a table. P-values are for p_Contra > 0.

emm <- emmeans(m.lat, ~ Trt | Time, transform = "response")
emm_show(emm)

| Trt | Time | prob | lower.HPD | upper.HPD | p.value |
| --- | --- | --- | --- | --- | --- |
| **50/50** | **30min** | **0.500** | **0.292** | **0.709** | **2.04e-05** |
| **BI** | **30min** | **0.751** | **0.561** | **0.910** | **2.22e-15** |
| **50/50** | **60min** | **0.501** | **0.294** | **0.713** | **2.18e-05** |
| **BI** | **60min** | **0.849** | **0.697** | **0.963** | **0.00e+00** |
| **50/50** | **180min** | **0.500** | **0.300** | **0.701** | **7.96e-06** |
| **BI** | **180min** | **0.815** | **0.650** | **0.943** | **0.00e+00** |

Plot of medians +- 95% HPDCI:


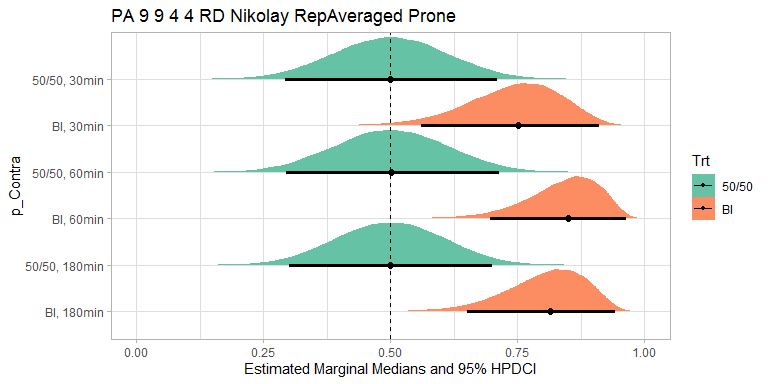


## p_Contra: Contrasts

Contrasts “between treatment and 50/50” as a table:

emmc <- contrast(emm, simple="Trt", method = "trt.vs.ctrl1")
emm_show(emmc)

| contrast | Time | estimate | lower.HPD | upper.HPD | p.value |
| --- | --- | --- | --- | --- | --- |
| BI - 50/50 | 30min | 0.246 | -0.033 | 0.519 | 8.42e-02 |
| **BI - 50/50** | **60min** | **0.341** | **0.084** | **0.594** | **9.11e-03** |
| **BI - 50/50** | **180min** | **0.309** | **0.053** | **0.557** | **1.73e-02** |

Contrasts “between treatment and 50/50” as a median +- 95% HPDCI:


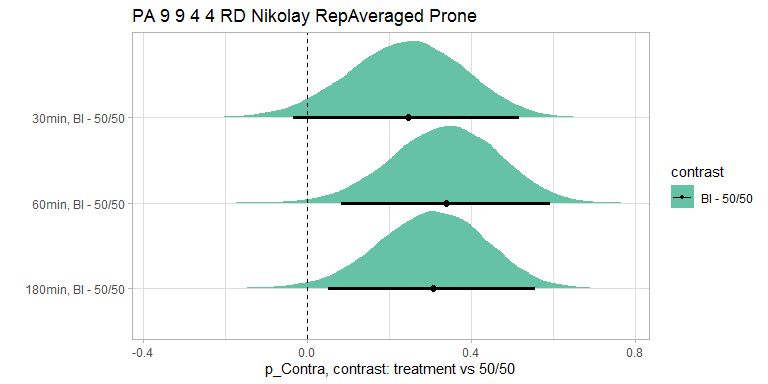


Contrasts “between treatment and 50/50” at Time=180min only as a table:

emm <- emmeans(m.lat, ~ Trt | Time, transform = "response", at = list(Time = c("180min")))
emmc <- contrast(emm, simple="Trt", method = "trt.vs.ctrl1")
emm_show(emmc)

| contrast | Time | estimate | lower.HPD | upper.HPD | p.value |
| --- | --- | --- | --- | --- | --- |
| **BI - 50/50** | **180min** | **0.309** | **0.053** | **0.557** | **1.73e-02** |

Contrasts “between treatment and 50/50” at Time=180min only as a median +- 95% HPDCI:


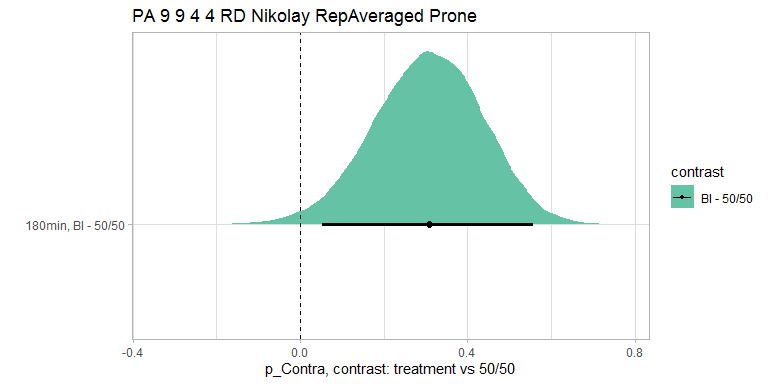


## p_Contra: MCMC conversion diagnostics

Posterior predictive check

tryCatch(print( plt[["p_Contra pp_check"]] <- pp_check(m.lat, nsamples = 11) ), error=function(e) "pp_check() failed")


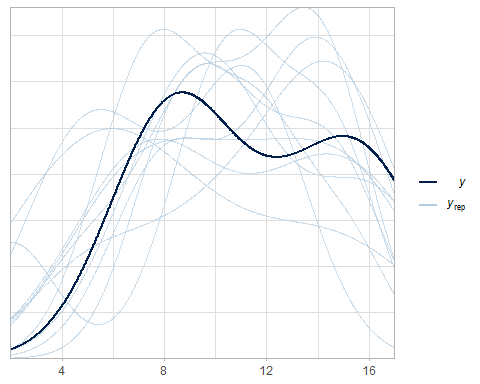


Autocorrelations

print( plt[["p_Contra ac"]] <- stan_ac(m.lat$fit) )


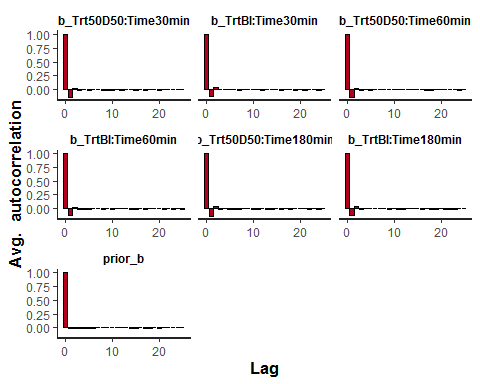


## Editable PowerPoint plots generated…
