# Supplementary material for: Left-right side-specific endocrine signaling complements neural pathways to mediate acute asymmetric effects of brain injury
Source: eLife. 2021 Aug 10;10:e65247. doi: 10.7554/eLife.65247 (PMC8354641; doi:10.7554/eLife.65247)
Supplement: Figure 2—source code 1. [file elife-65247-fig2-code1.zip › Figure 2CE - source code/RD9 TBI-SO Stud AI_Thr=log2(Contra_Ipsi).docx]

RD9, thresholds, P-values, 0.2s <= spikes <= 1s, AI

[Daniil.Sarkisyan@farmbio.uu.se](mailto:Daniil.Sarkisyan@farmbio.uu.se)

2019-08-05

## Read preprocessed RD9 data

rd_00read_data_06RD2019-01-23.R: reads timestamps, counts stikes, QC;

rd_03SC_AI_19_RD9.R: fits Bayesian multilevel model for the asymmetry index AI = log2( (1+Contra)/(1+Ipsi) ) using neuronal spikes counts at [0.2; 1] sec after stimulation (slow responses).

load(file="../HarmonizedData Nabe RD 2019-01-23/EMG-RD2019-01-23-data.RData")
load(file="AI_Threshold_RD9-2019-02-02.RData")

myname <- "RD9 TBI-SO Stud AI_Thr=log2(Contra_Ipsi)" # TBI vs Sham, merging OperationSide
#myname <- "RD9 LTBI-RTBI-SO Stud AI_Thr=log2(Contra_Ipsi)"
#myname <- "RD9 LTBI-RTBI-LSO-RSO Stud AI_Thr=log2(Contra_Ipsi)"
mymod <- fit[["AI.2.Thr"]] # AI.2.Thr , AI.3.Thr or AI.4.Thr

d <- emgThrLat %>% # AI = log2 Contra/Ipsi
 mutate( MSL = paste(Muscle, StimLocat, sep="."),
 MSL = factor(MSL, sort(unique(MSL))),
 Op3 = replace(paste0(OperationSide,Operation), Operation=="Sham", "Sham"),
 Op3 = factor(Op3, c("Sham","LeftTBI","RightTBI"), c("SO","LTBI","RTBI")) ) %>%
 droplevels(.)

The group sizes are (distinct rats only, same rats with different stimulation locations are counted as duplicates)

| Muscle | Sham | TBI |
| --- | --- | --- |
| EDL | 10 | 8 |
| Int | 9 | 17 |
| PL | 10 | 10 |
| ST | 7 | 7 |

## Model Overview

Model fit summary from brms:

## Family: student
## Links: mu = identity; sigma = identity; nu = identity
## Formula: AI_Threshold ~ Operation * Muscle + (1 || RatNo)
## Data: tmp (Number of observations: 84)
## Samples: 4 chains, each with iter = 40000; warmup = 20000; thin = 1;
## total post-warmup samples = 80000
##
## Group-Level Effects:
## ~RatNo (Number of levels: 29)
## Estimate Est.Error l-95% CI u-95% CI Rhat Bulk_ESS Tail_ESS
## sd(Intercept) 0.58 0.33 0.03 1.26 1.00 17761 28534
##
## Population-Level Effects:
## Estimate Est.Error l-95% CI u-95% CI Rhat Bulk_ESS
## Intercept 0.35 0.55 -0.72 1.43 1.00 43178
## OperationTBI -0.08 0.84 -1.73 1.57 1.00 37841
## MuscleInt -0.61 0.75 -2.09 0.88 1.00 49757
## MusclePL -0.07 0.71 -1.46 1.33 1.00 53159
## MuscleST -0.20 0.83 -1.84 1.44 1.00 53564
## OperationTBI:MuscleInt 0.81 1.06 -1.27 2.90 1.00 40726
## OperationTBI:MusclePL -1.20 1.10 -3.40 0.95 1.00 44440
## OperationTBI:MuscleST -1.91 1.23 -4.33 0.49 1.00 45859
## Tail_ESS
## Intercept 55772
## OperationTBI 52010
## MuscleInt 57527
## MusclePL 56070
## MuscleST 58770
## OperationTBI:MuscleInt 52758
## OperationTBI:MusclePL 56272
## OperationTBI:MuscleST 57744
##
## Family Specific Parameters:
## Estimate Est.Error l-95% CI u-95% CI Rhat Bulk_ESS Tail_ESS
## sigma 1.63 0.18 1.28 2.00 1.00 42793 40569
## nu 21.30 13.59 4.73 55.66 1.00 78622 49497
##
## Samples were drawn using sampling(NUTS). For each parameter, Eff.Sample
## is a crude measure of effective sample size, and Rhat is the potential
## scale reduction factor on split chains (at convergence, Rhat = 1).

Overlay of data points and model fit


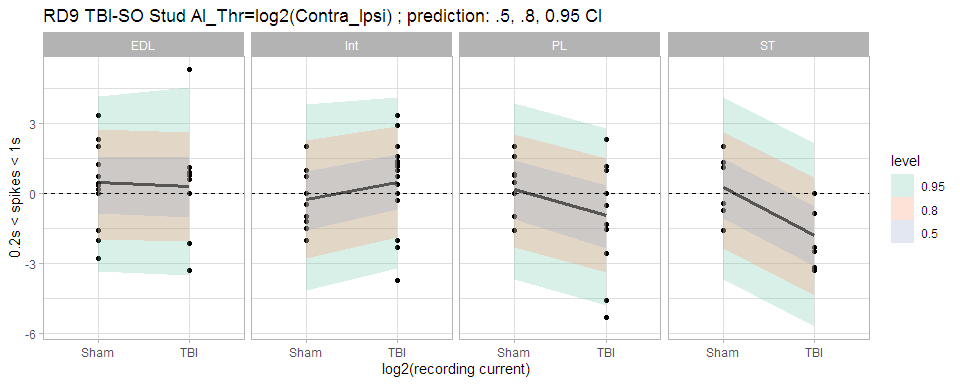


## Estimated model means

Estimated model means as a table:

emm <- emmeans(mymod, ~ Operation | Muscle)
# emm <- emmeans(mymod, ~ Op3 | Muscle)
# emm <- emmeans(mymod, ~ Operation*OperationSide | Muscle)
emm_show(emm)

| Operation | Muscle | emmean | lower.HPD | upper.HPD | p.value |
| --- | --- | --- | --- | --- | --- |
| Sham | EDL | 0.357 | -0.702 | 1.447 | 7.68e-01 |
| TBI | EDL | 0.268 | -0.997 | 1.496 | 8.89e-01 |
| Sham | Int | -0.256 | -1.440 | 0.917 | 8.92e-01 |
| TBI | Int | 0.478 | -0.370 | 1.340 | 4.74e-01 |
| Sham | PL | 0.285 | -0.763 | 1.351 | 8.38e-01 |
| TBI | PL | -0.999 | -2.219 | 0.224 | 2.05e-01 |
| Sham | ST | 0.157 | -1.202 | 1.497 | 9.67e-01 |
| **TBI** | **ST** | **-1.840** | **-3.169** | **-0.477** | **1.52e-02** |

Estimated model means as a median +- 95% QI (QI = quantile interval):


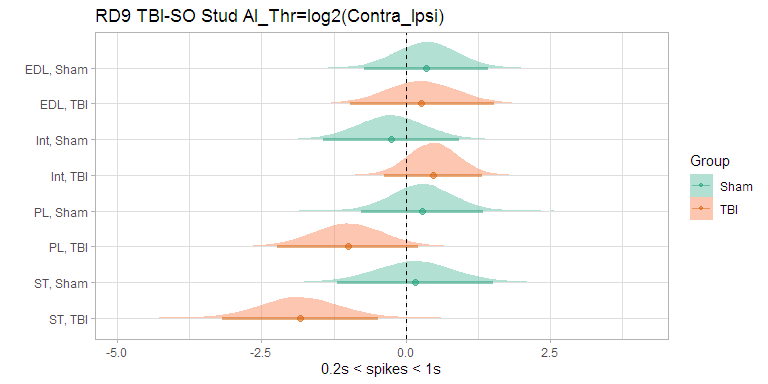


## Contrasts

Contrasts as a table:

emmc <- pairs(emm, simple="Operation", reverse=TRUE)
# emmc <- pairs(emm, simple="Op3", reverse=TRUE)
# emmc <- rbind( pairs(emm, simple="Operation", reverse=TRUE), pairs(emm, simple="OperationSide", reverse=TRUE))
emm_show(emmc)

| contrast | Muscle | estimate | lower.HPD | upper.HPD | p.value |
| --- | --- | --- | --- | --- | --- |
| TBI - Sham | EDL | -0.085 | -1.746 | 1.554 | 9.23e-01 |
| TBI - Sham | Int | 0.734 | -0.744 | 2.188 | 3.26e-01 |
| TBI - Sham | PL | -1.287 | -2.907 | 0.316 | 1.18e-01 |
| **TBI - Sham** | **ST** | **-1.992** | **-3.911** | **-0.106** | **4.01e-02** |

Contrasts TBI vs Sham as a median +- 95% QI (QI = quantile interval):


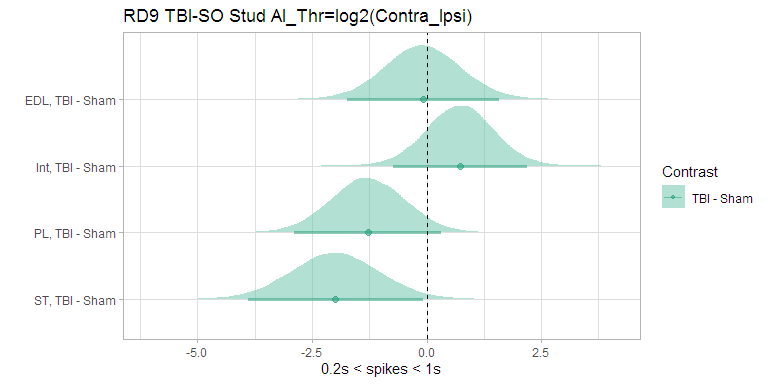


Contrasts Left operation vs Right operation as a median +- 95% QI (QI = quantile interval):

Contrasts of contrasts: “TBI vs Sham for Left-Right” is the same as “Left vs Right for TBI-Sham”

# emmc2c <- pairs(pairs(emm, simple="OperationSide", reverse=TRUE), simple="Operation", reverse=TRUE)
# emm_show(emmc2c)

TBI vs Sham as a median +- 95% QI (QI = quantile interval) of Left operation - Right operation:

## MCMC conversion diagnostics

Posterior predictive check

print( plt5 <- pp_check(mymod) )

## Using 10 posterior samples for ppc type 'dens_overlay' by default.


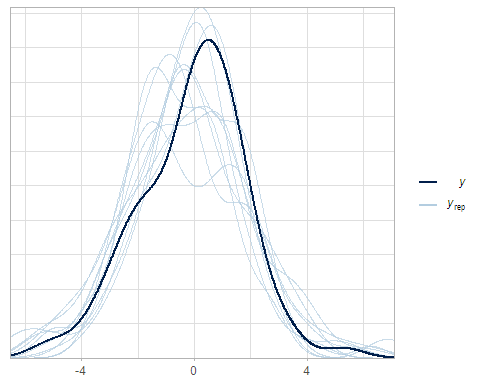


Autocorrelations

print( plt6 <- stan_ac(mymod$fit) )

## 'pars' not specified. Showing first 10 parameters by default.


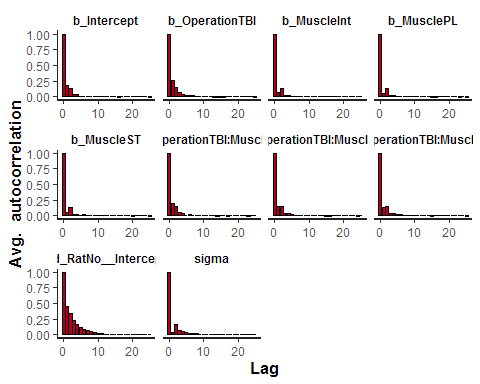


## Generate PowerPoint plots

Also export plots into the PowerPoint as editable objects instead of bitmaps
