# Supplementary material for: Left-right side-specific endocrine signaling complements neural pathways to mediate acute asymmetric effects of brain injury
Source: eLife. 2021 Aug 10;10:e65247. doi: 10.7554/eLife.65247 (PMC8354641; doi:10.7554/eLife.65247)
Supplement: Figure 2—source code 2. [file elife-65247-fig2-code2.zip › Figure 2DF - source code/RD9 TBI-SO Stud AI=log2(Contra_Ipsi).docx]

RD9, P-values, 0.2s <= spikes <= 1s, AI

[Daniil.Sarkisyan@farmbio.uu.se](mailto:Daniil.Sarkisyan@farmbio.uu.se)

2019-08-05

## Read preprocessed RD9 data

rd_00read_data_06RD2019-01-23.R: reads timestamps, counts stikes, QC;

rd_03SC_AI_19_RD9.R: fits Bayesian multilevel model for the asymmetry index AI = log2( (1+Contra)/(1+Ipsi) ) using neuronal spikes counts at [0.2; 1] sec after stimulation (slow responses).

load(file="../HarmonizedData Nabe RD 2019-01-23/EMG-RD2019-01-23-data.RData")
load(file="AI_RD9_2019-01-31.RData")

myname <- "RD9 TBI-SO Stud AI=log2(Contra_Ipsi)" # TBI vs Sham, merging OperationSide
#myname <- "RD9 LTBI-RTBI-SO Stud AI=log2(Contra_Ipsi)"
#myname <- "RD9 LTBI-RTBI-LSO-RSO Stud AI=log2(Contra_Ipsi)"
mymod <- fit[["AI.2.slow"]] # AI.2.slow , AI.3.slow or AI.4.slow

d <- emgLat %>% # AI = log2 Contra/Ipsi
#d <- emgHindpaw %>% # AI = log2 Left/right
 mutate( MSL = paste(Muscle, StimLocat, sep="."),
 MSL = factor(MSL, sort(unique(MSL))),
 Op3 = replace(paste0(OperationSide,Operation), Operation=="Sham", "Sham"),
 Op3 = factor(Op3, c("Sham","LeftTBI","RightTBI"), c("SO","LTBI","RTBI")),
 RC.log2 = log2(RecCurrent) ) %>%
 droplevels(.)

The group sizes are (distinct rats only, same rats with different stimulation locations are counted as duplicates)

| Muscle | Sham | TBI |
| --- | --- | --- |
| EDL | 10 | 8 |
| Int | 9 | 17 |
| PL | 10 | 10 |
| ST | 7 | 7 |

## Model Overview

Model fit summary from brms:

## Family: student
## Links: mu = identity; sigma = identity; nu = identity
## Formula: AI_0.2_1s ~ Operation * Muscle + RC.log2 * Muscle + (1 || RatNo)
## Data: tmp (Number of observations: 1344)
## Samples: 4 chains, each with iter = 40000; warmup = 20000; thin = 1;
## total post-warmup samples = 80000
##
## Group-Level Effects:
## ~RatNo (Number of levels: 29)
## Estimate Est.Error l-95% CI u-95% CI Rhat Bulk_ESS Tail_ESS
## sd(Intercept) 1.66 0.25 1.26 2.23 1.00 16640 26663
##
## Population-Level Effects:
## Estimate Est.Error l-95% CI u-95% CI Rhat Bulk_ESS
## Intercept -0.95 0.64 -2.21 0.31 1.00 20099
## OperationTBI 2.38 0.68 1.05 3.72 1.00 16744
## MuscleInt 1.15 0.45 0.27 2.03 1.00 32337
## MusclePL -2.36 0.64 -3.61 -1.12 1.00 32733
## MuscleST 1.44 1.08 -0.66 3.57 1.00 38729
## RC.log2 0.10 0.10 -0.10 0.30 1.00 31706
## OperationTBI:MuscleInt -3.85 0.25 -4.33 -3.36 1.00 47457
## OperationTBI:MusclePL -2.72 0.28 -3.28 -2.16 1.00 43765
## OperationTBI:MuscleST 0.37 0.30 -0.22 0.96 1.00 49788
## MuscleInt:RC.log2 0.02 0.13 -0.22 0.27 1.00 31218
## MusclePL:RC.log2 0.77 0.17 0.45 1.10 1.00 30778
## MuscleST:RC.log2 -0.28 0.24 -0.75 0.20 1.00 35660
## Tail_ESS
## Intercept 31759
## OperationTBI 26631
## MuscleInt 43563
## MusclePL 44139
## MuscleST 46597
## RC.log2 44831
## OperationTBI:MuscleInt 51555
## OperationTBI:MusclePL 52344
## OperationTBI:MuscleST 53675
## MuscleInt:RC.log2 41671
## MusclePL:RC.log2 41501
## MuscleST:RC.log2 44942
##
## Family Specific Parameters:
## Estimate Est.Error l-95% CI u-95% CI Rhat Bulk_ESS Tail_ESS
## sigma 1.34 0.05 1.22 1.43 1.00 41651 45347
## nu 18.37 10.51 6.82 46.52 1.00 41071 49345
##
## Samples were drawn using sampling(NUTS). For each parameter, Eff.Sample
## is a crude measure of effective sample size, and Rhat is the potential
## scale reduction factor on split chains (at convergence, Rhat = 1).

Overlay of data points and model fit


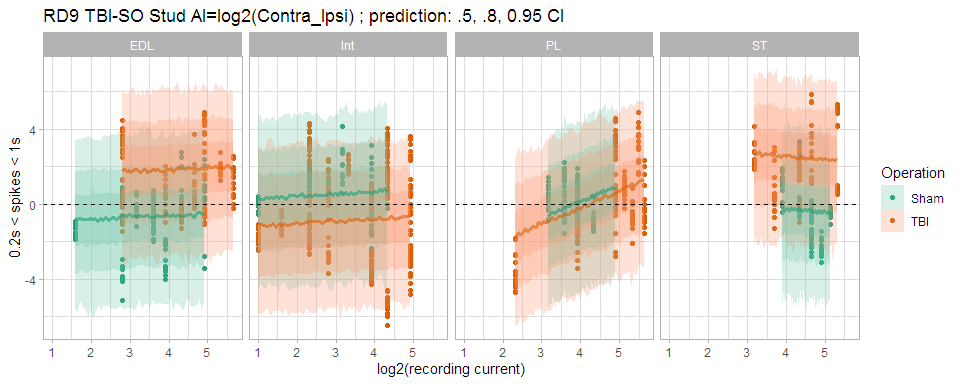


## Estimated model means

Estimated model means as a table:

emm <- emmeans(mymod, ~ Operation | Muscle)
# emm <- emmeans(mymod, ~ Op3 | Muscle)
# emm <- emmeans(mymod, ~ Operation*OperationSide | Muscle)
emm_show(emm)

| Operation | Muscle | emmean | lower.HPD | upper.HPD | p.value |
| --- | --- | --- | --- | --- | --- |
| Sham | EDL | -0.564 | -1.596 | 0.457 | 4.82e-01 |
| **TBI** | **EDL** | **1.818** | **0.990** | **2.655** | **3.56e-05** |
| Sham | Int | 0.675 | -0.373 | 1.711 | 3.65e-01 |
| TBI | Int | -0.787 | -1.592 | 0.012 | 1.03e-01 |
| Sham | PL | 0.076 | -0.943 | 1.102 | 9.87e-01 |
| TBI | PL | -0.262 | -1.112 | 0.598 | 7.97e-01 |
| Sham | ST | -0.186 | -1.289 | 0.891 | 9.29e-01 |
| **TBI** | **ST** | **2.560** | **1.691** | **3.415** | **1.08e-08** |

Estimated model means as a median +- 95% QI (QI = quantile interval):


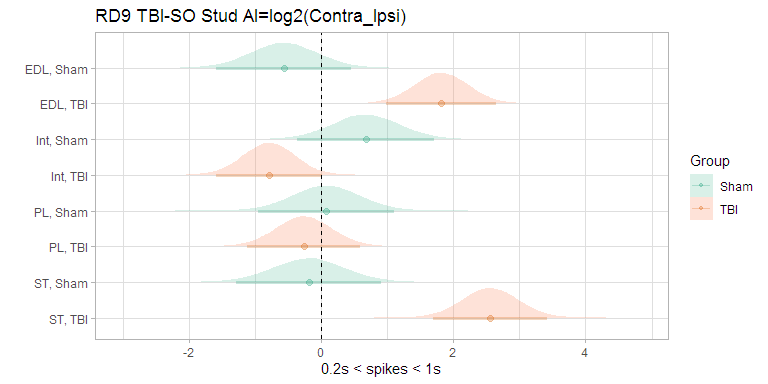


## Contrasts

Contrasts as a table:

emmc <- pairs(emm, simple="Operation", reverse=TRUE)
# emmc <- pairs(emm, simple="Op3", reverse=TRUE)
# emmc <- rbind( pairs(emm, simple="Operation", reverse=TRUE), pairs(emm, simple="OperationSide", reverse=TRUE))
emm_show(emmc)

| contrast | Muscle | estimate | lower.HPD | upper.HPD | p.value |
| --- | --- | --- | --- | --- | --- |
| **TBI - Sham** | **EDL** | **2.379** | **1.080** | **3.743** | **4.39e-04** |
| **TBI - Sham** | **Int** | **-1.463** | **-2.782** | **-0.159** | **2.81e-02** |
| TBI - Sham | PL | -0.339 | -1.644 | 1.039 | 6.22e-01 |
| **TBI - Sham** | **ST** | **2.745** | **1.419** | **4.128** | **6.37e-05** |

Contrasts TBI vs Sham as a median +- 95% QI (QI = quantile interval):


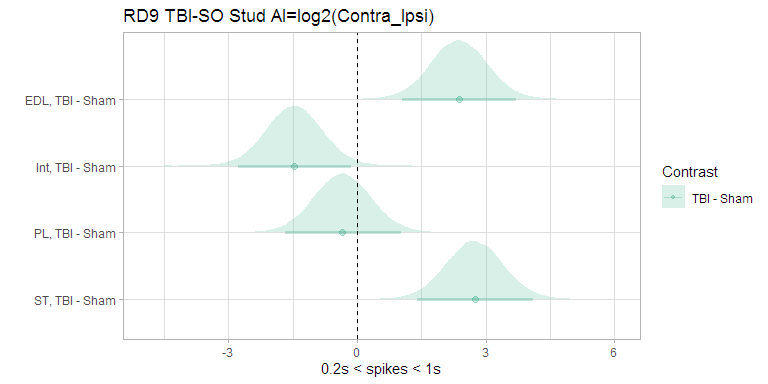


Contrasts Left operation vs Right operation as a median +- 95% QI (QI = quantile interval):

Contrasts of contrasts: “TBI vs Sham for Left-Right” is the same as “Left vs Right for TBI-Sham”

# emmc2c <- pairs(pairs(emm, simple="OperationSide", reverse=TRUE), simple="Operation", reverse=TRUE)
# emm_show(emmc2c)

TBI vs Sham as a median +- 95% QI (QI = quantile interval) of Left operation - Right operation:

## MCMC conversion diagnostics

Posterior predictive check

print( plt5 <- pp_check(mymod) )

## Using 10 posterior samples for ppc type 'dens_overlay' by default.


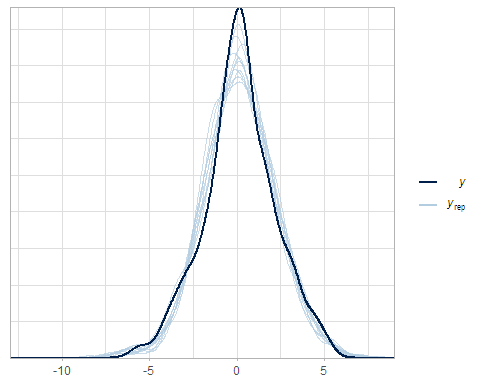


Autocorrelations

print( plt6 <- stan_ac(mymod$fit) )

## 'pars' not specified. Showing first 10 parameters by default.


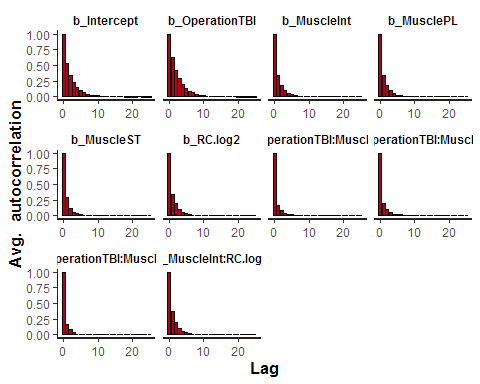


## Generate PowerPoint plots

Also export plots into the PowerPoint as editable objects instead of bitmaps
